# No evidence of altered language laterality in people who stutter across different brain imaging studies of speech and language

**DOI:** 10.1093/braincomms/fcae305

**Published:** 2024-09-13

**Authors:** Birtan Demirel, Jennifer Chesters, Emily L Connally, Patricia M Gough, David Ward, Peter Howell, Kate E Watkins

**Affiliations:** Department of Experimental Psychology, Wellcome Centre for Integrative Neuroimaging, University of Oxford, Oxford OX2 6GG, UK; Department of Experimental Psychology, Wellcome Centre for Integrative Neuroimaging, University of Oxford, Oxford OX2 6GG, UK; Department of Experimental Psychology, Wellcome Centre for Integrative Neuroimaging, University of Oxford, Oxford OX2 6GG, UK; School of Psychology, University College Dublin, Dublin DN720/PCS2, Ireland; School of Psychology & Clinical Language Sciences, University of Reading, Reading RG6 6AL, UK; Experimental Psychology, Psychology & Language Sciences, University College London, London WC1E 6BT, UK; Department of Experimental Psychology, Wellcome Centre for Integrative Neuroimaging, University of Oxford, Oxford OX2 6GG, UK

**Keywords:** stuttering, speech disorder, language lateralization, brain imaging, fMRI

## Abstract

A long-standing neurobiological explanation of stuttering is the incomplete cerebral dominance theory, which refers to competition between two hemispheres for ‘dominance’ over handedness and speech, causing altered language lateralization. Renewed interest in these ideas came from brain imaging findings in people who stutter of increased activity in the right hemisphere during speech production or of shifts in activity from right to left when fluency increased. Here, we revisited this theory using functional MRI data from children and adults who stutter, and typically fluent speakers (119 participants in total) during four different speech and language tasks: overt sentence reading, overt picture description, covert sentence reading and covert auditory naming. Laterality indices were calculated for the frontal and temporal lobes using the laterality index toolbox running in Statistical Parametric Mapping. We also repeated the analyses with more specific language regions, namely the pars opercularis (Brodmann area 44) and pars triangularis (Brodmann area 45). Laterality indices in people who stutter and typically fluent speakers did not differ, and Bayesian analyses provided moderate to anecdotal levels of support for the null hypothesis (i.e. no differences in laterality in people who stutter compared with typically fluent speakers). The proportions of the people who stutter and typically fluent speakers who were left lateralized or had atypical rightward or bilateral lateralization did not differ. We found no support for the theory that language laterality is reduced or differs in people who stutter compared with typically fluent speakers.

## Teaser

Demirel *et al*. report no significant differences in language laterality in children and adults who stutter compared with typically fluent speakers based on analysis of functional MRI data across different overt and covert language tasks. These findings challenge the incomplete cerebral dominance theory of stuttering.

## Graphical Abstract

**Graphical Abstract:**
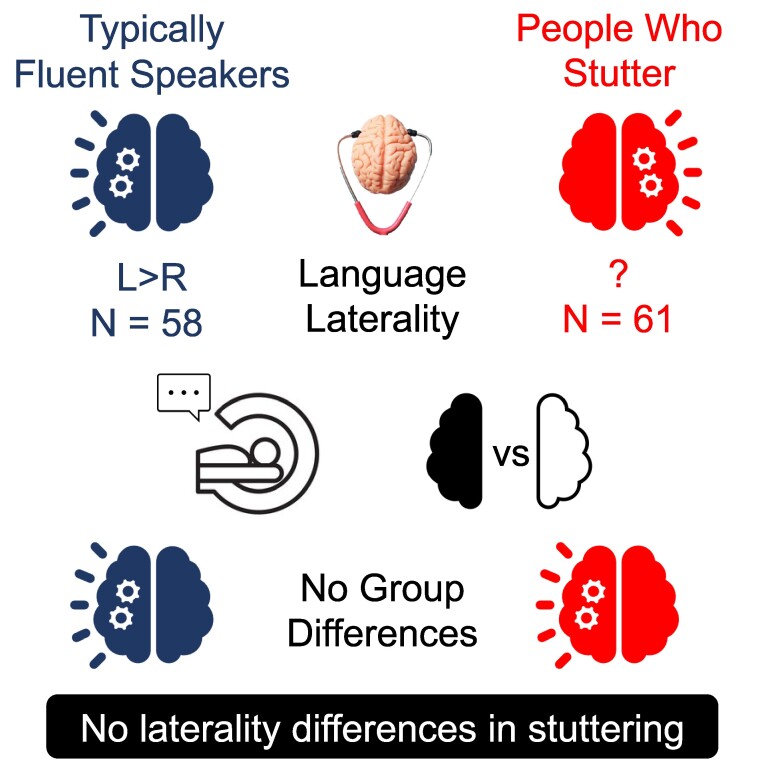

## Introduction

Persistent developmental stuttering is a speech fluency disorder affecting ∼1% of people worldwide that appears to occur in all languages. The onset of stuttering is typically between ages 2 and 4 years,^1^ which corresponds with the rapid development of language skills, such as increase in the average length of utterance.^2^ The occurrences of stuttering are highly variable both within and across individuals. For example, the frequency of stuttered syllables is affected by different factors, including the complexity of grammar and the length of the planned utterance.^2-4^ Language proficiency is typically unaffected in people who stutter (PWS)^5,6^; PWS know what they want to say, but the flow of speech is disrupted by repetitions, prolongations and blocks.

Language is perhaps the most lateralized cognitive function: showing primarily left hemisphere involvement for around 96% of right-handers and 70% of left-handers; while the rest, 4% of right-handers and 30% of left-handers have what is called atypical lateralization, indicating either bilateral or primarily right hemisphere involvement.^7^ The underlying neurophysiology of stuttering is not yet fully understood; however, one popular neurological explanation of stuttering is the incomplete cerebral dominance theory, which suggests altered patterns of hemispheric specialization and competition between hemispheres for ‘dominance’ over handedness and speech.^8,9^ Although scientific interest in the incomplete cerebral dominance theory in stuttering declined over recent decades, it persists as an explanation frequently given by PWS themselves. For example, some PWS describe themselves as being naturally left-handed, but due to the forced use of the right hand, say on school entry, they started to stutter. It is argued that excessive use of the nondominant hand weakens the dominant hemisphere over time. This can cause conflict between hemispheres, leading to altered language lateralization, which refers to the dominance of one cerebral hemisphere over the other in managing language functions.^9^

The first functional neuroimaging meta-analysis in stuttering appeared to support the incomplete cerebral dominance theory by suggesting a tendency towards rightward lateralization in PWS relative to controls during speech production tasks.^10^ Overactivation in the right inferior frontal cortex is commonly reported in stuttering during speech production tasks, particularly activity of the right frontal operculum and anterior insula^11,12^ extending to the orbitofrontal cortex.^13^ Moreover, the overactivity in the right hemisphere is described as being ‘normalized’ with speech therapy resulting in typical left-lateralized activity.^13-15^ Patterns of activity and timing of responses in auditory networks during passive listening tasks also differ between PWS and controls.^16-19^ Sato *et al.*^18^ using near-infrared spectroscopy documented diverse laterality in PWS during passive listening to pairs of syllables that included either a phonemic or prosodic contrast, which typically produce left- and right-lateralized patterns of activity, respectively, in fluent speakers. Notably, none of the adults, school-aged children or pre-school children who stuttered showed significant expected patterns of lateralization for either phonemic or prosodic contrasts, and some showed the reverse pattern.

Even though there are consistent findings of overactivity in the right hemisphere in the functional imaging literature, it still needs to be clarified whether these findings reflect an altered pattern of lateralization. To demonstrate this, it is necessary to statistically compare activity in the two hemispheres during different speech and language tasks. This approach typically uses a laterality index (LI) calculated using the formula LI = (L − R)/(L + R) to compare the functional activity between two hemispheres. Positive LIs indicate leftward lateralization and negative LIs indicate rightward lateralization and LIs between −0.2 ≤ LI ≤ 0.2 indicate weak lateralization, sometimes called ‘bilateral’.^20^ The values entered into the formula for the left and right hemispheres reflect the amount of activity in a given brain region. This is expressed as per cent signal change from a baseline or other contrast, or in terms of the number of activated voxels above a certain threshold. This threshold is sometimes referred to as ‘activated tissue volume,’ or it can be the sum of the values of these voxels, which is typically a statistic that reflects the strength of a voxel’s correlation with the task. LIs calculated in this way from brain imaging data can be strongly influenced by individual differences in brain activation and noise levels, the statistical threshold employed and statistical outliers.^21^ For example, above a particular threshold, no active voxels might remain in the nondominant hemisphere leading to LI = 1 for the dominant hemisphere, which is biologically implausible. The LI toolbox^20^ running in Statistical Parametric Mapping (SPM, www.fil.ion.ucl.ac.uk/spm/software/spm12/) avoids threshold dependency by calculating LI values across multiple threshold levels and uses a bootstrapping approach to reduce the effect of outliers. The LI toolbox has been successfully employed to measure laterality because of these advantages.^22^

To address the incomplete cerebral dominance theory in stuttering, we calculated laterality indices for four different speech and language tasks performed using functional MRI (fMRI) in PWS and typically fluent speakers (TFS). Our analyses of these laterality indices were used to target two main questions: (i) Are there any differences between PWS and TFS in functional lateralization across speech and language tasks? (ii) Does functional lateralization vary across tasks in PWS or TFS or both?

## Materials and methods

### Participants

We combined data from several cohorts of children and adults who stutter who were scanned over the past decade using fMRI during different speech and language tasks. Following exclusion for excessive movement during scanning (details below), useable data were available in 119 participants, 61 PWS (*M*_age_ = 29 years, range = 14–54 years) and 58 TFS (*M*_age_ = 28 years, range = 14–53 years). The groups were not significantly different in terms of age: *t*(117) = 0.60, *P* = 0.55. A *χ*^2^ analysis showed that the groups did not differ in terms of the distribution of handedness (*χ*^2^ = 2.23, *P* = 0.13) or gender (*χ*^2^ = 2.22, *P* = 0.14). One study with 38 participants (23 PWS, 15 TFS) included only right-handed male participants; therefore, we repeated the *χ*^2^ analysis assessing the distribution of handedness after excluding data from this study and again found no difference between groups in terms of the distribution of handedness (*χ*^2^ = 3.40, *P* = 0.06). For the PWS group, stuttering ranged in severity from very mild to very severe (9–46, median 26) according to the Stuttering Severity Instrument.^23^ At the time of the scan, stuttering severity was assessed as very mild in 8, mild in 11, moderate in 25, severe in 11 and very severe in 6 PWS. The largest subset of participants had performed one of four slightly different versions of overt sentence reading: 56 PWS and 53 TFS. Smaller subsets had completed tasks involving overt picture description (16 PWS and 18 TFS) and two covert speech tasks involving sentence reading (12 PWS and 12 TFS) and auditory naming (12 PWS and 16 TFS). Table 1 shows the distribution of handedness and sex between groups.

**Table 1:** Distribution of handedness and sex of groups across tasks

Handedness, gender and age	PWS						TFS					
	*N*	Handedness		Gender		*M*_age_	*N*	Handedness		Gender		*M*_age_
		L	R	W	M			L	R	W	M	
Overt sentence reading	56	7	49	10	46	28	53	2	51	13	40	28
Overt picture description	16	2	14	4	12	30	18	2	16	5	13	32
Covert sentence reading	12	3	9	3	9	30	12	1	11	3	9	29
Covert auditory naming	12	3	9	3	9	30	16	1	15	6	10	27

PWS, people who stutter; TFS, typically fluent speakers; L, left; R, right; W, women; M, men; *M*_age_, mean age (in years).

### Tasks

**Figure 1:**
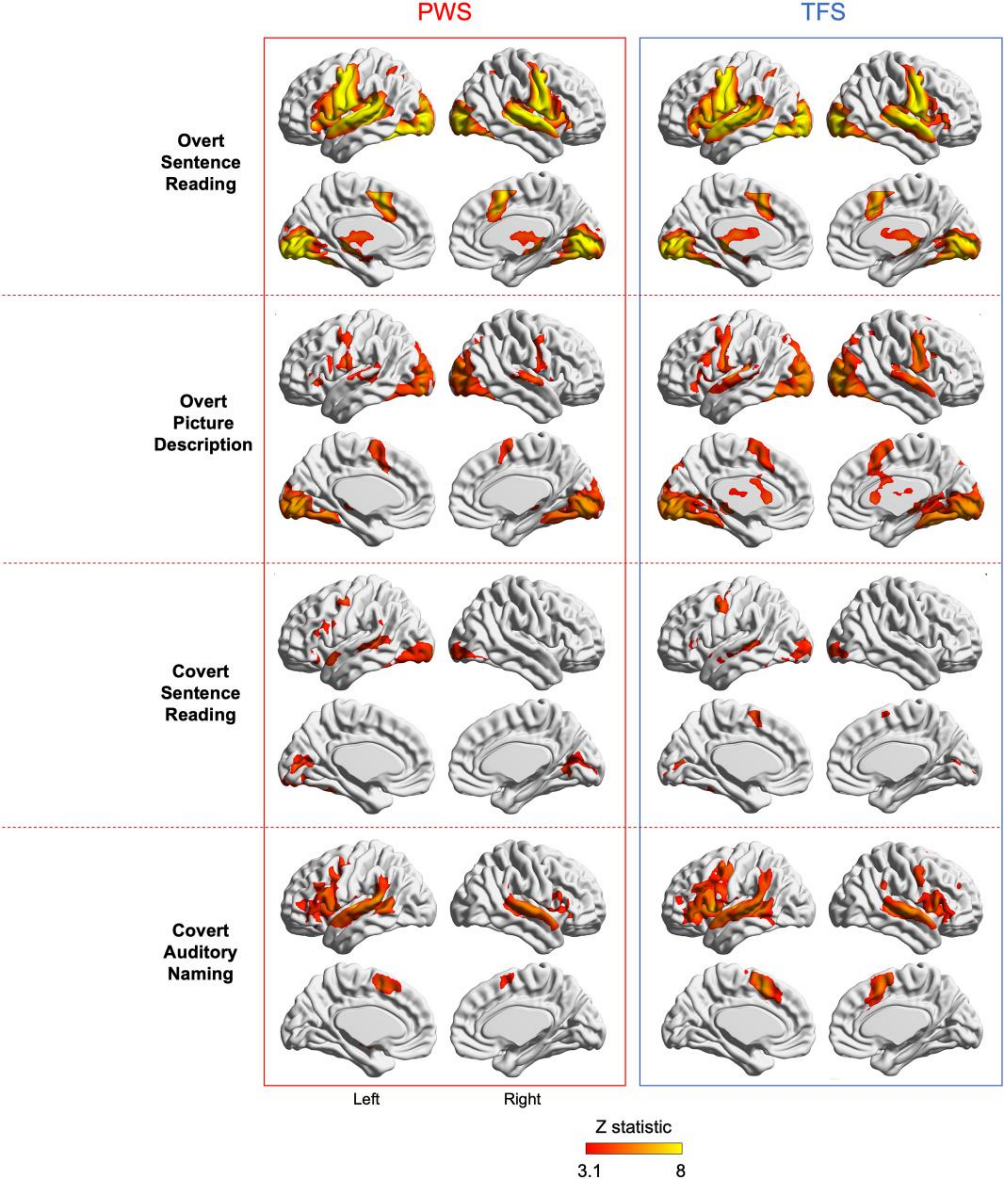
**Average brain activity for groups of PWS and TFS during four different tasks.** Patterns of brain activity were similar in the two groups. The coloured statistical maps are of *Z*-statistic images thresholded using clusters determined by *Z* > 3.1 and a corrected cluster significance threshold of *P* = 0.05 overlaid on the MNI-152/Talairach surface template using BrainNet Viewer.^25^ Medial and lateral surfaces of the left and right hemispheres are shown. PWS: people who stutter are on the left of the image; TFS: typically fluent speakers are on the right. The sample sizes for each task were as follows: overt sentence reading: PWS = 56, TFS = 53; overt picture description: PWS = 16, TFS = 18; covert sentence reading: PWS = 12, TFS = 12; covert auditory naming: PWS = 12, TFS = 16.

The tasks analysed included overt sentence reading combined from different studies that have been conducted in our laboratories, overt picture description, covert sentence reading and covert auditory naming tasks (Fig. 1). Overt speech refers to audible production of words/sentences, while covert speech refers to silent production of words/sentences with no articulation. A sparse-sampling design^24^ was used in the overt speaking tasks, in which participants spoke during the silent period between scans. This ensures that the imaging data are not confounded with speech–movement-related artefacts and reduces the possible fluency-enhancing effect of the scanner noise. It also allowed participants to hear the speech they produced. In contrast, continuous acquisition protocols of typical echo-planar imaging were used for the covert speech tasks. Details of the scanners used and scanning parameters are provided in Supplementary Table 1 with reference to published versions of these studies.

#### Overt sentence reading

During all versions of the sentence reading task, participants saw a sentence printed in white on a black screen. In some participants (34), a scene corresponding to the sentence was also visible. All participants read the sentence out loud and could hear themselves speaking over headphones during the silent intervals between scan measurements. The baseline conditions consisted of viewing a row of letter X’s that replaced all other letters in a sentence (37 participants), or sentences written in the Farsi alphabet (38 participants), which was unreadable by participants, or a fixation cross (34 participants). Activity during sentence reading was contrasted with the baseline to reveal the brain areas involved in reading aloud, which involves visual word recognition, semantic and syntactic processing, phonological assembly, speech articulation and monitoring of self-produced speech.

#### Overt picture description

The task was to describe a picture that appeared on the screen using the participant’s own words and spoken out loud. As for overt sentence reading, participants could hear their speech during the silent intervals between scans. The task required conceptualization of the scene, lexical selection and retrieval, phonological and syntactic planning, speech articulation and monitoring of self-produced speech. A fixation cross was used as a baseline for the picture description task.^26^

#### Covert sentence reading

Participants read sentences in their imagined speech. In contrast to the overt sentence reading task, reading a sentence covertly does not involve execution of articulatory movements or sensory feedback. This task was contrasted with a row of X’s presented on the screen.

#### Covert auditory naming

Participants heard a short phrase (e.g. ‘bees make it’) over headphones and were asked to generate a word in response (e.g. ‘honey’) in their imagined speech. The task requires decoding the phonological and semantic information of the auditory input, lexical search and retrieval, and phonological form encoding to generate a word associated with the semantic description.^27^ A silent baseline and reversed speech baseline conditions were used for the auditory naming task.

### fMRI data analysis

The fMRI data were analysed at the single-subject level using FEAT (FMRI Expert Analysis Tool part of the FMRIB Software Library).^28^ The echo-planar images were motion corrected, smoothed using an 8 mm full-width at half-maximum smoothing kernel, undistorted using field maps where available and registered to the individual’s T_1_-weighted structural image using boundary-based registration. These individual T_1_-weighted images were then registered to the MNI-152 template using FMRIB’s non-linear registration tool (FNIRT embedded in FSL). The six motion regressors from the motion correction were included as covariates of no interest. For continuous imaging sequences (the two covert tasks), the task design was convolved with the double-gamma haemodynamic response function and temporal derivatives were included in the general linear model. For sparse-sampling sequences, convolution with the haemodynamic response function and temporal derivatives were turned off.

Data from five additional scans from the PWS group were excluded from further data analysis due to excessive head movements during scanning. Exclusion criteria were set concerning the mean displacements of head movements being larger than the widest voxel dimension (i.e. >4 mm) relative to subsequent time points or a warning from the MCFLIRT motion correction tool^29^ concerning high levels of motion.

### Quantifying laterality indices

The statistical maps for individual participants were registered to the MNI-152 standard space using their individual T_1_-weighted structural images (see above) and transferred to the SPM software (http://fil.ion.ucl.ac.uk/spm/) for use with the laterality toolbox.^21^ The LI toolbox uses a weighted bootstrapping algorithm to create LI values from the fMRI data by obtaining voxel values for both the left and right hemispheres. First, a vector of voxel values is created from the masked and thresholded input image. Then the algorithm resamples the voxel values 100 times each from the left and right regions of interest (ROIs). All possible LI combinations are calculated from these samples 10 000 times. This procedure is repeated for 20 different threshold intervals (from 0 to the maximum threshold value). Finally, all LIs are plotted on a histogram, and only the central 50% is used as the final LI measure to reduce the influence of outliers. In addition, the LI toolbox produces a weighted mean from the results obtained at different thresholds, which ensures that voxels showing a higher correlation with the task have more impact on the result. Therefore, LI values acquired at a higher threshold are given more weight in the final LI-weighted mean value.

ROIs were chosen based on masks of the frontal and temporal lobes, as these include the regions typically activated by the language tasks. Activity from voxels in the medial wall was excluded from the LI calculations since it is difficult to determine the hemispheric origin of activity at this location due to smoothing (standard exclusion mask: midline ± 5 mm). In separate analyses, we also measured the LI values in smaller ROIs, namely the pars opercularis (BA44) and pars triangularis (BA45) of the inferior frontal gyrus, to provide a more granular analysis. Masks were generated using the Juelich atlas within FSL software, for the BA44 and BA45 brain regions with a threshold set at 30%.

### Statistical analysis

**Figure 2:**
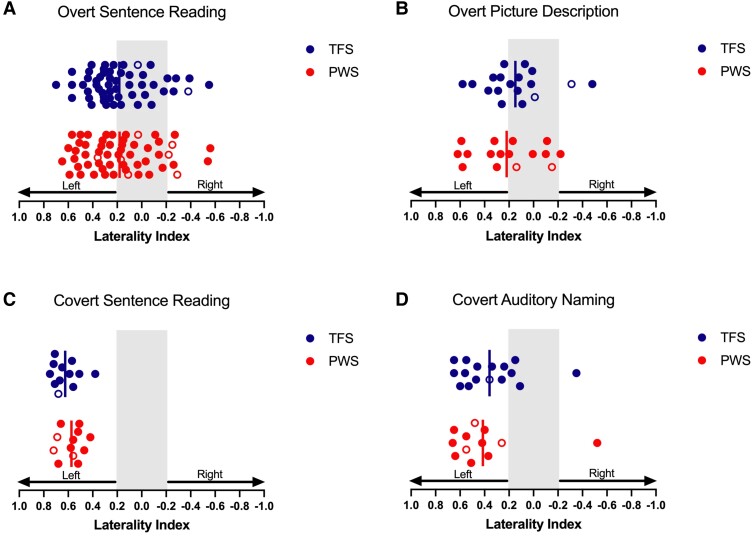
**Laterality indices in PWS and TFS based on activity in the frontal lobes.** Solid vertical lines represent group means. The grey area represents LI values between −0.2 and 0.2, which are considered not lateralized. PWS: people who stutter; TFS: typically fluent speakers. The open circles indicate left-handedness in both groups. We performed Bayesian *t*-tests to test the differences in LI-weighted values between PWS and TFS across four different speech and language tasks. The Bayesian independent samples *t*-tests yielded a BF in favour of the null hypothesis of (**A**) 4.92 for overt sentence reading (PWS = 56, TFS = 53), (**B**) 2.41 for overt picture description (PWS = 16, TFS = 18), (**C**) 1.55 for covert sentence reading (PWS = 12, TFS = 12) and (**D**) 2.56 for covert auditory naming (PWS = 12, TFS = 16) tasks.

Initial inspection of the data suggested no group differences in terms of laterality indices (see Fig. 2 below); therefore, we selected a Bayesian approach in order to weigh the evidence in favour of the null relative to the alternative hypothesis. We performed Bayesian *t*-tests to address our first research question to test whether there are differences in LI-weighted values between PWS and the TFS group for four different speech and language tasks. We reported the Bayesian factor 01 value, which refers to the ratio of the ‘likelihood of data given the null hypothesis/likelihood of data given the alternative hypothesis’. In alignment with Jeffreys’^30^ guidelines, we considered a Bayes factor (BF) falling within the range of 1–3 as indicative of anecdotal evidential support, while a BF ranging from 3 to 10 signifies a moderate level of evidential support. A BF surpassing 10, on the other hand, indicates robust evidential support. We also categorized data sets as showing typical patterns of leftward laterality (>0.2) compared with atypical (<−0.2 right or bilateral, −0.2 to 0.2 LIs), to determine whether the distribution of laterality in individuals differed between groups using *χ*^2^ analysis.

To compare laterality indices among the different tasks, linear mixed model regression analyses were performed. All Bayesian and *χ*^2^ tests were carried out using the software JASP,^31^ and we used RStudio^32^ to perform linear mixed model regression analyses.

## Results

### Brain activity during tasks: group averages

We looked at the brain activity of each group relative to the baseline condition for each task based on a cluster-forming threshold of *Z* > 3.1 and an extent threshold of *P* < 0.05 (corrected for family-wise error). Both groups showed a broad range of activity in brain regions expected to be activated by speech and language (Fig. 1 and Table 2).

**Table 2:** Laterality indices across tasks for the frontal and temporal lobes, BA44 and BA45

Region × task	PWS				TFS			
	*N*	Mean LI + SEM	Typ	Atyp	*N*	Mean LI + SEM	Typ	Atyp
Frontal lobes								
Overt sentence reading	56	0.18 ± 0.03	28	28	53	0.18 ± 0.03	31	22
Overt picture description	16	0.21 ± 0.07	9	7	18	0.14 ± 0.06	8	10
Covert sentence reading	12	0.57 ± 0.02	12	0	12	0.62 ± 0.03	12	0
Covert auditory naming	12	0.41 ± 0.09	10	2	16	0.36 ± 0.06	12	4
Temporal lobes								
Overt sentence reading	56	0.12 ± 0.04	29	27	53	0.13 ± 0.04	24	29
Overt picture description	16	−0.06 ± 0.06	4	12	18	0.00 ± 0.05	6	12
Covert sentence reading	12	0.56 ± 0.06	11	1	12	0.58 ± 0.04	12	0
Covert auditory naming	12	0.23 ± 0.08	9	3	16	0.24 ± 0.05	11	5
BA44								
Overt sentence reading	56	0.18 ± 0.05	30	26	53	0.20 ± 0.05	28	25
Overt picture description	16	0.40 ± 0.07	12	4	18	0.34 ± 0.07	13	5
Covert sentence reading	12	0.59 ± 0.03	12	0	12	0.51 ± 0.09	11	1
Covert auditory naming	12	0.50 ± 0.08	11	1	16	0.41 ± 0.06	14	2
BA45								
Overt sentence reading	56	0.04 ± 0.06	24	32	53	0.13 ± 0.05	25	28
Overt picture description	16	0.34 ± 0.11	12	4	18	0.19 ± 0.09	10	8
Covert sentence reading	12	0.51 ± 0.09	11	1	12	0.45 ± 0.11	10	2
Covert auditory naming	12	0.43 ± 0.09	10	2	16	0.40 ± 0.10	14	2

LI, laterality indices’ weighted mean; SEM, standard error of mean; Typ, participants who show typical pattern of leftward laterality; Atyp, participants who show right or bilateral laterality.

During the overt sentence reading task, large portions of the primary motor cortex, premotor cortex, presupplementary motor area extending to anterior cingulate cortex, superior temporal lobe, the thalamus, ventral occipito-temporal cortex and lobule VI of the cerebellum were significantly activated bilaterally in the PWS group but somewhat more extensively on the left. The TFS group exhibited strikingly similar brain activity patterns to the PWS group.

The overt picture description task evoked significant activity in premotor and primary motor cortex, the presupplementary motor area, superior temporal gyrus, thalamus, ventral occipito-temporal cortex and the anterior lobe of the cerebellum, all bilaterally with additional activation of the left inferior frontal gyrus in both groups that was more extensive in the TFS group.

The covert sentence reading task evoked significant activity in inferior frontal cortex, primary motor cortex (at the level of the face representation), superior temporal gyrus and mid fusiform gyrus in the left hemisphere, and in lateral and medial occipital cortex bilaterally in both groups. There was additional activity in the cerebellar vermis in PWS and presupplementary motor area in the TFS group.

During the covert auditory naming task, there was significant activity in the inferior frontal gyrus bilaterally but more extensively on the left, the presupplementary motor area, the superior temporal gyrus and dorsal striatum bilaterally, and in the right anterior and posterior lobes of the cerebellum in both groups. In the PWS, there was significant activity in the primary motor cortex on the left and this was seen bilaterally in the TFS group; TFS also had activity in the left anterior lobe of the cerebellum.

### LIs based on task-evoked activity in the frontal lobes

#### Overt sentence reading task

The results of our LI calculations for the frontal lobe masks for the sentence reading task are shown in Table 2 and Fig. 2. The sentence reading task was completed by the largest number of participants combined across four different versions of the task. A Bayesian independent samples *t*-test (two sided) revealed a BF of 4.92, indicating moderate evidence in support of the null hypothesis (LIs are similar between PWS and TFS groups) rather than the alternative hypothesis (LIs differ between PWS and TFS). *χ*^2^ statistics found that the number of individuals in each group who showed the typical pattern of leftward laterality compared with atypical (right or bilateral LIs) did not differ (*χ*^2^ = 0.79, *P* = 0.37; see Table 2). It is worth noting that the mean LI for each group falls in the range considered indicative of weak laterality or bilateral (Table 2), indicating that the tasks are not reliably strongly lateralized, though individual values ranged from −0.56 to 0.65 in PWS and −0.55 to 0.70 in TFS.

#### Overt picture description, covert sentence reading and covert auditory naming

In different subsets of the overall sample, we also analysed LIs for the overt picture description task, covert sentence reading task and covert auditory naming task (Table 2 and Fig. 2). Bayesian independent samples *t*-test analyses revealed BFs of 2.41, 1.55 and 2.56, respectively, for these tasks, indicating anecdotal evidence in support of the null hypothesis (no group differences for each task). On the basis of these findings in smaller subsets of the sample, we can neither reject nor support the null hypothesis. *χ*^2^ analyses found no group differences regarding the number of typically or atypically lateralized individuals in the overt picture description task (*χ*^2^ = 0.47, *P* = 0.49), covert sentence reading task (all participants were left lateralized) and covert auditory naming task (*χ*^2^ = 0.28, *P* = 0.59).

#### Laterality effect of different tasks

Inspection of Fig. 2 indicates that, for the frontal lobes, the two covert tasks (covert sentence reading and auditory naming) were more robustly lateralized at both the group and the individual levels relative to the pattern of lateralization seen for the overt tasks (overt sentence reading and picture description). To test this quantitatively, we employed linear mixed models with overt tasks as a fixed effect. Group (PWS and TFS) was entered into the model as interactions for this fixed effect with intercepts of subjects as our random effect. The model revealed that LIs were significantly more left lateralized for covert tasks (*N* = 51, LI = 0.49, SE = 0.03) compared with overt tasks (*N* = 143, LI = 0.18, SE = 0.02) (*β* = −0.39, *t* = −6.33, *P* < 0.001). There were no significant interactions with the group.

### LIs based on task-evoked activity in the temporal lobes

#### Overt sentence reading

**Figure 3:**
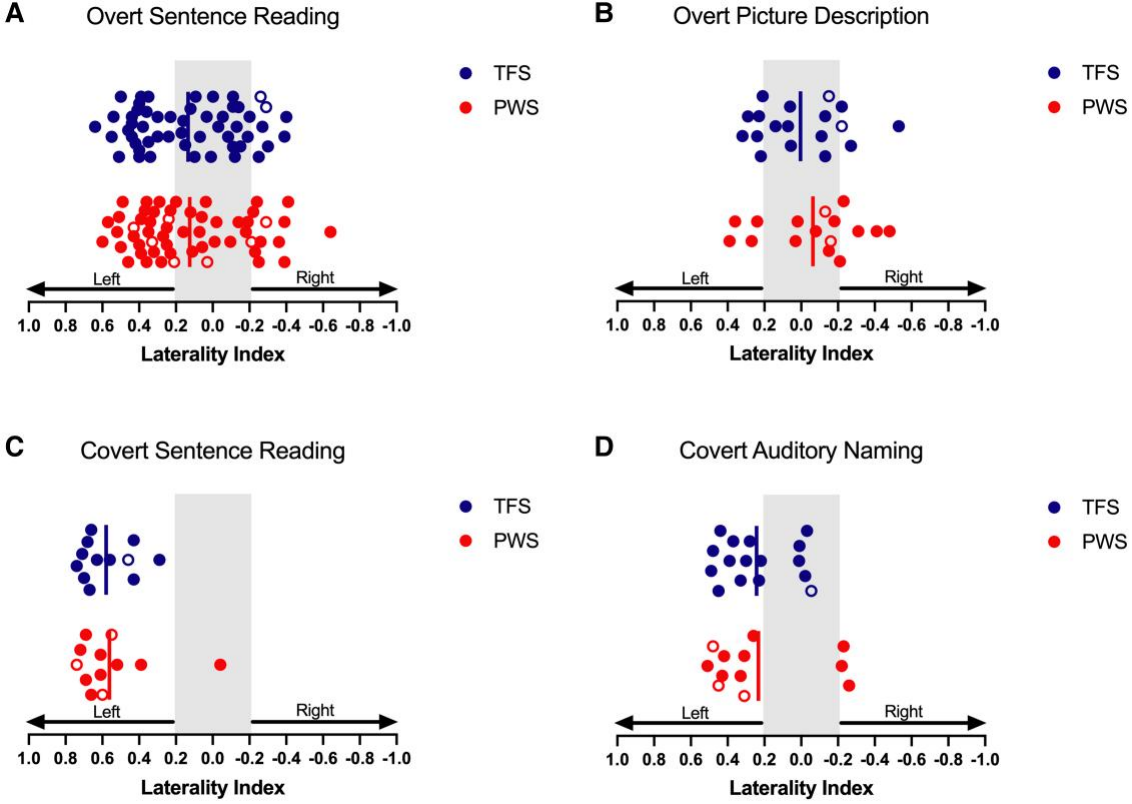
**Laterality indices in PWS and TFS based on activity in the temporal lobes (see Fig. 2 for details).** Results from the Bayesian independent samples *t*-tests indicated a BF in support of the null hypothesis of (**A**) 4.87 for overt sentence reading (PWS = 56, TFS = 53), (**B**) 2.36 for overt picture description (PWS = 16, TFS = 18), (**C**) 2.61 for covert sentence reading (PWS = 12, TFS = 12) and (**D**) 2.80 for covert auditory naming (PWS = 12, TFS = 16) tasks.

For the temporal lobe masks, a Bayesian independent samples *t*-test revealed a BF of 4.87, indicating moderate evidence in support of the null hypothesis. The *χ*^2^ analysis gave the same result as the analysis of frontal lobes and verified that the groups did not differ regarding the number of typically or atypically lateralized individuals (*χ*^2^ = 0.46, *P* = 0.50). As above, the mean LIs indicate this is not a robustly lateralized task at the group level, though again the range of values indicates considerable variation among individuals (Fig. 3 and Table 2).

#### Overt picture description, covert sentence reading and covert auditory naming

For the temporal lobe masks, we examined LIs for the overt picture description task, covert sentence reading task and covert auditory naming task (Fig. 3 and Table 2). Bayesian independent samples *t*-tests found no support for the alternative hypothesis with BFs of 2.36, 2.61 and 2.80, respectively, indicating anecdotal evidence in support of the null hypothesis. Furthermore, *χ*^2^ analyses found the same results as for the frontal lobe analyses, which confirmed that the groups did not differ in terms of the number of typically or atypically lateralized individuals in the overt picture description task (*χ*^2^ = 0.28, *P* = 0.9), covert sentence reading task (*χ*^2^ = 1.04, *P* = 0.30) and covert auditory naming task (*χ*^2^ = 0.13, *P* = 0.72) (see Table 2).

#### Laterality effect of different tasks

The same linear mixed model analysis as above was performed on the data from the temporal lobe masks to test differences in lateralization between covert and overt tasks (Fig. 3). Similarly to the above, the model revealed that the covert tasks (*N* = 51, LI = 0.40, SE = 0.04) showed significantly more robust left lateralization than overt tasks (*N* =143, LI = 0.09, SE = 0.02) (*β* = −0.30, *t* = −4.32, *P* < 0.001).

### LIs for task-evoked activity in pars opercularis (BA44) and pars triangularis (BA45)

We analysed the LI values within more focal ROIs, specifically the pars opercularis (BA44) and pars triangularis (BA45) of the inferior frontal gyrus (Table 2). This approach was adopted to facilitate a more detailed examination since these regions in the left hemisphere correspond to ‘Broca’s area’ and are usually robustly activated by speech and language tasks. Again, we found similar results suggesting no differences between groups in LIs (see Supplementary Figs. 1 and 2). For BA44, data from the overt sentence reading task (which has the largest sample size) provided moderate evidence in favour of the null hypothesis with the BF of 4.82. For the other tasks, we found only anecdotal evidence in favour of the null: overt picture description (BF = 2.69), covert sentence reading (BF = 2.24) and covert auditory naming (BF = 2.05). For BA45, we only found anecdotal evidence in favour of the null hypothesis for each of the four tasks; overt sentence reading (BF = 2.91), overt picture description (BF = 2.08), covert sentence reading (BF = 2.49) and covert auditory naming tasks (BF = 2.78).

*χ* ^2^ analyses also revealed no significant differences between the groups in terms of the number of participants in each group who were typically or atypically lateralized: BA44 in overt sentence reading (*χ*^2^ = 0.01, *P* = 0.93), overt picture description (*χ*^2^ = 0.03, *P* = 0.85), covert sentence reading (*χ*^2^ = 1.04, *P* = 0.30) and covert auditory naming (*χ*^2^ = 0.12, *P* = 0.72); BA45 in overt sentence reading (*χ*^2^ = 0.20, *P* = 0.65), overt picture description (*χ*^2^ = 1.40, *P* = 0.23), covert sentence reading (*χ*^2^ = 0.38, *P* = 0.53) and covert auditory naming (*χ*^2^ = 0.10, *P* = 0.75) tasks (see Table 2).

## Discussion

Since the pioneering findings of Paul Broca, it is well documented that most people rely more on their left hemisphere than their right to use language. In the current study, we investigated the theory as to whether PWS have reduced hemispheric specialization compared with TFS. We looked at data obtained across different language and speech tasks, namely overt sentence reading, overt picture description, covert sentence reading and covert auditory naming that we obtained in different fMRI studies. Pooling data across these different versions allowed us to investigate one task (overt sentence reading) in a relatively large sample of 56 PWS compared with 53 TFS. We analysed LI-weighted means for task-evoked activity in frontal and temporal lobes and repeated the same analyses for portions of the inferior frontal gyrus involved in language processing, namely the pars opercularis (BA44) and pars triangularis (BA45) regions. We did not exclude stuttering epochs from the overt speaking data in this analysis since it is possible that stuttering evokes right hemisphere activity. The inclusion of stuttering epochs therefore made it more likely we would detect a rightward pattern of lateralization in PWS should one exist.

Our main findings are as follows: (i) there was no evidence in support of the idea that PWS are differently lateralized relative to people who are typically fluent and some anecdotal and moderate evidence in support of the idea that they are equally lateralized; (ii) leftward lateralization was most robustly observed for the covert tasks relative to the overt tasks and this effect was seen in both groups.

### No differences in laterality between PWS and TFS

The idea that PWS are differently lateralized for language has a long history dating back to Samuel Orton (1927),^9^ who first proposed this idea, and it is a persistent ‘urban’ myth believed by many individuals with lived experience of stuttering. The support for this myth comes from two main sources of information: (i) it has been suggested that left-handedness may be a risk factor for stuttering and that it occurs more frequently in PWS than in the general population,^33,34^ which is often assumed to be associated with more rightward lateralization for language in the brain^8,9^, and (ii) some early observations and neuroimaging studies proposed atypical hemispheric activity during stuttered speech that shifted to the left when individuals spoke fluently.^15,35-37^

First, our groups of PWS and TFSs did not differ in the distribution of left- and right-handedness, while it is important to note that one of the studies with 38 participants (23 PWS, 15 TFS) recruited only right-handed male participants. Nevertheless, the distribution of handedness between our groups remained stable, even when data from that study were excluded from our handedness analysis. Therefore, our data are consistent with evidence indicating that stuttering is independent from handedness.^38-40^ We find no support for the idea that PWS are more likely to be left-handed than TFS, nor that their handedness alters the typical pattern of left hemispheric cerebral dominance for speech and language.

In our main findings, we found that PWS and TFS show equivalent levels of language lateralization across a range of tasks. The means for groups of PWS and TFS were very similar, and they had very similar distributions of participants categorized as typically (left) or atypically (right or bilaterally) lateralized. This pattern of results was seen for our large ROI analyses, where we compared task-evoked activity in the frontal and temporal lobes, as well as in our analyses of more focused ROIs in the inferior frontal cortex. The results of our statistical analyses found no support for the idea that PWS are differently lateralized on average relative to people who are fluent speakers; in fact, in some cases, we found moderate support for the hypothesis that they are not differently lateralized. These results are compatible with a magnetoencephalography study that compared language lateralization in pre-school children who stutter and controls during picture naming tasks.^41^ The authors reported that the language was mostly left lateralized in both groups over frontal, temporal and parietal regions without significant differences between groups.

The lack of a rightward lateralization during speech production in PWS might appear inconsistent with the robust finding of right hemisphere overactivity reported in several studies of PWS.^11-13,15^ This right hemisphere overactivity in the frontal operculum/anterior insula was described as one of the ‘neural signatures’ of stuttering.^10^ In our frontal lobe analysis, both the frontal operculum and the anterior insula were included in the mask, and the BA44 mask included at least a portion of the frontal operculum. Any overactivity in these areas would have contributed to the data being compared between the two hemispheres in the calculation of laterality indices. The apparent discrepancy in these findings is explained by two key features of the laterality calculations. First, voxel values between the two hemispheres were compared across a range of statistical thresholds, so that even sub-threshold (in a whole-brain group comparison) data from both hemispheres contributed to the overall calculation. Second, activity was summed for supra-threshold voxels over large regions (whole lobes in the first instance) rather than specific portions of gyri identified as unilaterally overactive in PWS. In this latter case, high voxel values in focal locations, such as the right anterior insula, could have been offset by similar voxel values distributed more widely across different portions of left frontal lobe when sampled across the whole region.

Another apparent discrepancy with our findings comes from reports that therapeutic interventions for stuttering demonstrate the potential to enhance neural activity within the left hemisphere of the brain^42^ or shift the balance of activity from the right hemisphere to the left during speech production.^13,15^ However, neither of these previous studies statistically compared the activity between two hemispheres in PWS and controls. Focal areas of overactivation in one hemisphere during speech tasks in PWS might reflect a range of functions unrelated to language, such as inhibition, compensation or error responses (e.g. see Neef *et al.*^12^). Furthermore, as explained above, the unilateral overactivity may be due to statistical thresholding, leading to the conclusion that there is no activity in one hemisphere because it is only visible sub-threshold and emphasizing the need for statistical comparisons between hemispheres.

To our knowledge, there is only one study that found support for the idea that PWS are differently lateralized for language, which also investigated language laterality directly. Using near-infrared spectroscopy, Sato *et al*.^18^ reported laterality during passive listening of syllable pairs, which included either a phonemic contrast (e.g. /itta/ vs. /itte/) or prosodic contrast (e.g. /itta/ vs. /itta?/). Specifically, adults, school-aged children and pre-school children who stutter did not show the expected leftward lateralization for processing the phonemic contrast, whereas controls showed a pattern of leftward and rightward lateralization for phonemic and prosodic contrasts, respectively.^18^ Although the results of a direct comparison of language laterality between PWS and controls differ from our findings, it must be considered that this study involved passive listening to words rather than production.

Studies on structural hemispheric differences in PWS can offer valuable insights for interpreting functional activity. For instance, some earlier structural imaging studies in PWS reported lower leftward (or more rightward) asymmetry in the planum temporale, which was correlated with stuttering severity.^43^ However, analysis of planum temporale asymmetry with a large population overlapping with the participants reported here found no differences between PWS and TFS and also no relationship with handedness.^44^

### LIs are more strongly left lateralized for the covert tasks

In our findings, covert language tasks were significantly more lateralized compared with overt tasks. This finding is consistent with another fMRI study that reported more robust language lateralization for covert language tasks than overt ones.^45^ The reason may be that the cortical motor areas that send hundreds of commands to dozens of muscles bilaterally during overt speech production are not involved in covert speech. When the motor cortex is heavily involved in overt articulation, perhaps this bilateral pattern of task-related activity reduces laterality measured by methods that include these areas. As seen in our overt tasks (see Fig. 1), pre- and post-central gyri are reliably activated bilaterally during overt speaking tasks compared with a passive baseline task (meta-analysis from PET and fMRI studies^46^). It is worth noting, however, that the analysis of the activity in BA44 and BA45 ROIs, which do not include data from the pre- and post-central gyri, also show stronger lateralization for the covert than the overt tasks (see Supplementary Figs. 1 and 2).

Another possible reason for stronger laterality during covert tasks in our findings needs to be highlighted. Both tasks (covert sentence reading and auditory naming) involved continuous data acquisition during imaging. In contrast, the overt speech production tasks were carried out using sparse sampling to allow participants to hear themselves during the task and to avoid potential head movement artefacts due to speech production during acquisition of the imaging data. Sparse sampling is feasible because the haemodynamic response is slow, only peaking some 4–6 s after the event; hence, speech production can occur during a relatively long window of silence (6–10 s across different studies) before the measurement that takes 2–3 s occurs.^24^ Sparse-sampling fMRI is the most effective way of reducing motion-related noise in the images due to overt speech production. Furthermore, sparse-sampling acquisitions are demonstrably better than continuous imaging for detecting auditory responses in temporal lobe cortex.^47,48^ However, to match the power of continuous imaging using simple block designs, sparse-sampling acquisitions need to be longer.^49^ They are also less sensitive to variations in the haemodynamic response within individuals and between them. Variations in task design (event versus block designs), timing (short or long delays in measurement) and in analysis methods (convolution with the haemodynamic response function versus finite impulse response models) hamper direct comparisons of the sensitivity of sparse-sampling relative to continuous acquisitions.^50^ It remains unclear, however, why laterality should differ between sparse-sampled and continuous acquisition since all factors affecting the sensitivity of the measurement should affect both hemispheres equally. Nevertheless, with our current data sets, we cannot disentangle possible causes of our finding that covert tasks were more strongly lateralized than overt ones since this factor is confounded with the measurement difference.

### Limitations

Laterality indices allow us to compare the activity of thousands of voxels between two hemispheres in a single metric; however, useful information may be lost with this approach. LI scores deriving from fMRI results are limited in temporal resolution, which is not informative for possible differences in lateralization milliseconds before the speech starts. For example, Neef *et al.*^51^ used transcranial magnetic stimulation during a verb generation task to record motor-evoked potentials from the tongue. They reported atypical cortical excitability patterns between left and right tongue motor cortical areas in adults who stutter milliseconds before speech onset. The current fMRI study would not be able to detect these dynamic differences in cortical excitability because of the relatively slow signal of the haemodynamic response.^52^

In comparing activity between hemispheres, choices need to be made regarding the ROIs. The advantage of large ROIs, such as whole hemispheres or lobes, is that spatial variation in language processing among participants will be captured. The disadvantage of large regions is that many of the voxels included do not show task-evoked activity. ROI selections were made relying on masks of the frontal and temporal lobes, as these encompass the areas where language tasks usually elicit activity. In addition, we quantified the LI values within more focal ROIs, specifically the pars opercularis (BA44) and pars triangularis (BA45) situated within the inferior frontal gyrus, thereby affording a finer-grained examination.

## Conclusion

Previous fMRI findings consistently reported an overactive right hemisphere in stuttering during speech tasks but did not statistically compare the functional activity between hemispheres. Therefore, they do not provide direct evidence for altered hemispheric specialization in PWS during language production. Here, our results close that gap by statistically comparing the functions of two hemispheres to test the altered hemispheric specialization theory with a threshold-independent laterality analysis. Our findings indicated no difference in the hemispheric specialization in frontal and temporal regions of PWS compared with TFS while performing four different speech and language tasks. We also reported that covert tasks were substantially more lateralized than overt tasks for both groups. These data were obtained using continuous imaging, whereas the overt tasks were carried out using sparse sampling. Therefore, task choice and data acquisition may be important factors to consider when measuring laterality.

## Supplementary Material

fcae305_Supplementary_Data

## Data Availability

The data sets analysed during the current study are available in the OSF. This includes laterality indices, demographics and stuttering severity scores for each participant.
