# Supplementary material for: No evidence of altered language laterality in people who stutter across different brain imaging studies of speech and language
Source: Brain Commun. 2024 Sep 13;6(5):fcae305. doi: 10.1093/braincomms/fcae305 (PMC11430911; doi:10.1093/braincomms/fcae305)
Supplement: fcae305_Supplementary_Data [file fcae305_supplementary_data.pdf]

Laterality indices for task-evoked activity in Pars Opercularis (BA44)

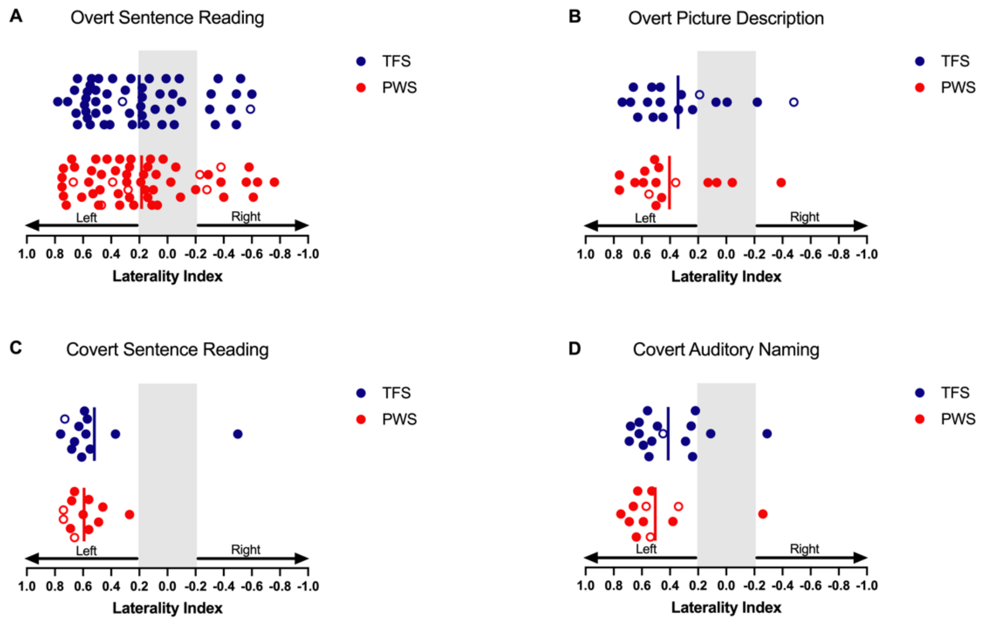

Supplementary Figure 1: Laterality indices in people who stutter and typically fluent speakers based on activity in Pars Opercularis. Solid vertical lines represent group means. The grey area represents LI values between -0.2 and 0.2, which are considered not lateralised. PWS: People who stutter are the red circles; TFS: Typically fluent speakers are the blue circles. The circles indicate left-handedness in both groups. The analysis using the Bayesian independent samples t-tests indicated a Bayes factor in support of the null hypothesis of: (A) 4.82 for Overt Sentence Reading (PWS = 56, TFS = 53), (B) 2.69 for Overt Picture Description (PWS = 16, TFS = 18), (C) 2.24 for Covert Sentence Reading (PWS = 12, TFS = 12), and (D) 2.05 for Covert Auditory Naming (PWS = 12, TFS = 16) tasks.

Laterality indices for task-evoked activity in Pars Triangularis (BA45)

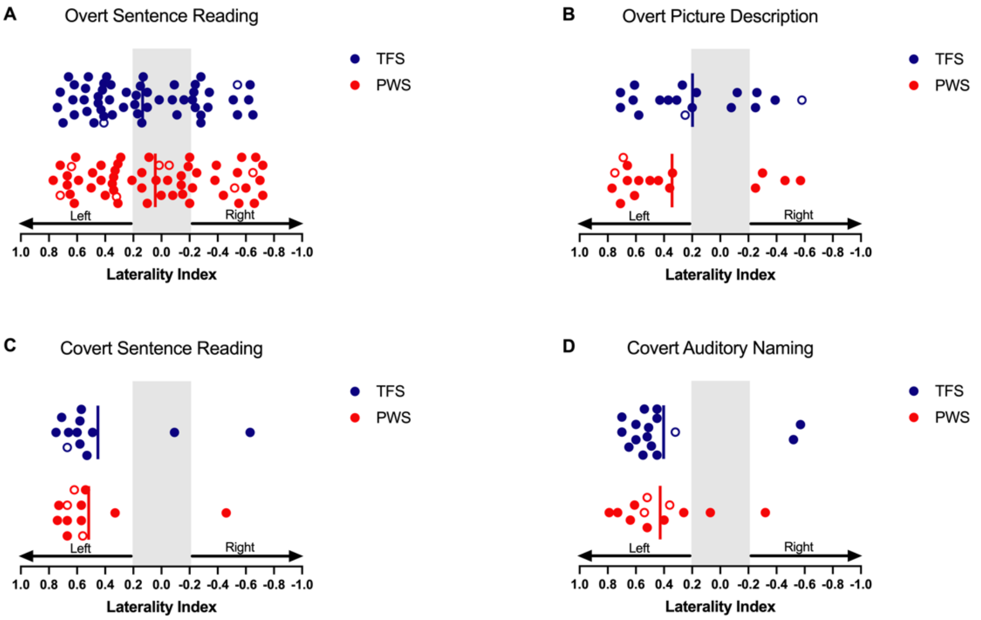

Supplementary Figure 2: Laterality indices in people who stutter and typically fluent speakers based on activity in Pars Triangularis (see Supplementary Figure 1 for details). The Bayesian independent samples t-tests generated a Bayes factor in support of the null hypothesis of: (A) 2.91 for Overt Sentence Reading (PWS = 56, TFS = 53), (B) 2.08 for Overt Picture Description (PWS = 16, TFS = 18), (C) 2.49 for Covert Sentence Reading (PWS = 12, TFS = 12), and (D) 2.78 for Covert Auditory Naming (PWS = 12, TFS = 16) tasks. PWS: People who stutter; TFS: Typically fluent speakers.

Supplementary Table 1. Scan details

Task	Baseline	PWS	TFS	Scanner	TE (ms)	TR/TA (s)	Slices (N)	Thick-ness (mm)	In-plane (mm)
Overt sentence reading (NAF/DAF)[1]	Row Xs	10	10	3T Varian-Siemens	30	10 / 3	32	4	4 x 4
Overt sentence reading (NAF/DAF)[2]	Row Xs	8	11	3T Siemens Trio	30	10 / 3	50	3	3 x 3
Overt sentence reading (NAF)[3]	Farsi script	23	15	3T Siemens Trio	30	9 / 2	38	3.5	3 x 3
Overt sentence reading / Picture description[4]	Fixation	16	18	3T Siemens Trio[6]	30	9 / 2	32	4	3 x 3
Covert Sentence Reading / Covert Reading & listening / Passive Listening[2,]	Row Xs	12	12	3T Siemens Trio	30	2.4/2.4	40	3	3 x 3
Covert Auditory Naming[2,5]	Reversed Speech / Fixation	12	16	1.5T Siemens Sonata	50	3/3	35	4	3 x 3

Supplementary references

[1] Watkins KE, Smith SM, Davis S, Howell P. Structural and functional abnormalities of the motor system in developmental stuttering. Brain. 2008;131(1):50-59. doi:10.1093/brain/awm241

[2] Unpublished data

[3] Chesters et al., 2021 (preprint): doi:https://doi.org/10.31219/osf.io/8st3j

[4] Connally EL, Ward D, Pliatsikas C, et al. Separation of trait and state in stuttering. Hum Brain Mapp. 2018;39(8):3109-3126. doi:https://doi.org/10.1002/hbm.24063

[5] Badcock NA, Bishop DVM, Hardiman MJ, Barry JG, Watkins KE. Co-localisation of abnormal brain structure and function in specific language impairment. Brain Lang. 2012;120(3):310-320. doi:https://doi.org/10.1016/j.bandl.2011.10.006

[6] Different Trio to the one used for the other tasks

NAF = normal auditory feedback; DAF = delayed auditory feedback; TE = Echo time (ms); TR = Repetition time (s); TA = acquisition time (s); acquisition is delayed in sparse sampling designs to allow a silent interval between acquisitions resulting in a longer TR (= delay + TA). If TA = TR then imaging was continuous.
